# A New Logging-While-Drilling Method for Resistivity Measurement in Oil-Based Mud †

**DOI:** 10.3390/s20041075

**Published:** 2020-02-16

**Authors:** Yongkang Wu, Baoping Lu, Wei Zhang, Yandan Jiang, Baoliang Wang, Zhiyao Huang

**Affiliations:** 1 State Key Laboratory of Industrial Control Technology, College of Control Science and Engineering, Zhejiang University, Hangzhou 310027, China; yk.wu@zju.edu.cn (Y.W.); ydjiang@zju.edu.cn (Y.J.); zy_huang@zju.edu.cn (Z.H.); 2 Sinopec Research Institute of Petroleum Engineering, Beijing 100101, China; lubp.sripe@sinopec.com (B.L.); zhangwei.sripe@sinopec.com (W.Z.); 3 State Key Laboratory of Shale Oil and Gas Enrichment Mechanisms and Effective Development, Beijing 100101, China

**Keywords:** resistivity logging, oil-based mud (OBM), logging-while-drilling (LWD), capacitively coupled contactless conductivity detection (C^4^D) technique, inductive coupling principle

## Abstract

Resistivity logging is an important technique for identifying and estimating reservoirs. Oil-based mud (OBM) can improve drilling efficiency and decrease operation risks, and has been widely used in the well logging field. However, the non-conductive OBM makes the traditional logging-while-drilling (LWD) method with low frequency ineffective. In this work, a new oil-based LWD method is proposed by combining the capacitively coupled contactless conductivity detection (C^4^D) technique and the inductive coupling principle. The C^4^D technique is to overcome the electrical insulation problem of the OBM and construct an effective alternating current (AC) measurement path. Based on the inductive coupling principle, an induced voltage can be formed to be the indirect excitation voltage of the AC measurement path. Based on the proposed method, a corresponding logging instrument is developed. Numerical simulation was carried out and results show that the logging instrument has good measurement accuracy, deep detection depth and high vertical resolution. Practical experiments were also carried out, including the resistance box experiment and the well logging experiment. The results of the resistance box experiment show that the logging instrument has good resistance measurement accuracy. Lastly, the results of the well logging experiment indicate that the logging instrument can accurately reflect the positions of different patterns on the wellbore of the experimental well. Both numerical simulation and practical experiments verify the feasibility and effectiveness of the new method.

## 1. Introduction

Reservoir evaluation plays an important role in the exploration and development of oil and gas. With the increase of unconventional wells in the drilling operations such as high angle wells and horizontal wells, logging-while-drilling (LWD) technology has become an important research aspect in the well logging field. LWD technology enables practitioners to measure the formation parameters for geological analysis while drilling.

Reservoir drilling is generally carried out in water-based mud (WBM) due to its convenience, low cost and little pollution to the environment [1]. However, with the development of LWD, WBM is no longer a good choice [1,2]. On the one hand, to achieve the fine reservoir evaluation in WBM environment, the expensive and quite time-consuming full-hole coring has to be adopted [1]. On the other hand, with the increase of deepwater wells and unconventional wells, WBM is no longer applicable because of the high temperature and high pressure resulted from these kinds of wells [2].

Due to the advantages of high temperature resistance, good lubricity and low reservoir damage, oil-based mud (OBM) is more suitable for most of deepwater wells and unconventional wells than WBM [3]. It can improve drilling efficiency and decrease operation risks. Thus, it has been widely used in the well logging field [4]. However, because the continuous phase of the OBM is oil, the resistivity of the OBM is usually million times the resistivity of the WBM resistivity [5]. It means the traditional measurement method with low frequency is no longer feasible in the OBM environment.

In view of the non-conductive OBM, new methods for solving the electrical insulation problem of the OBM have been proposed [2,4,6,7,8,9,10,11,12,13,14,15,16,17,18,19]. There are four main categories. The first method is to research and develop a conductive OBM system that possesses not only good lubricity and stability as the conventional OBM has, but also low resistivity comparable to that of WBM [7]. To increase the conductivity of the OBM significantly, additives such as conductive solid particles [8], polar base oil [9], oil soluble ionic salts [10], specified ionic liquids [11], nanomaterials [12,13,14,15] are required to be added into the OBM. With the conductive OBM, the traditional LWD method becomes still effective. However, this method is limited by the high cost of early research-development and the environmental consideration on the toxic additives. The second method is to make a knife-like electrode which is sharp enough to cut through the mud cake, so that a conductive path will be established between the electrode and the formation, and the conventional low-frequency measurement method becomes still applicable [16,17]. However, this method becomes infeasible in the complex wellbore conditions including the thick mud attachment, irregular wall, spiral and deviated well. The third method is called the four-terminal measurement, which is to utilize the potential difference between the two vertical offset voltage electrodes for calibrating the formation resistivity in an alternating current field [18]. This method has the advantages of large measurement range and good adaptability of the mud cake thickness. However, there are several disadvantages accompanying this method, such as low resolution and limited pad structure. The fourth method is based on the capacitive coupling principle [2,4,6,19]. In this method, a current-carrying electrode is used to emit a high-frequency current to the formation and then the resistivity information of the formation can be obtained by measuring the voltage of the voltage-sensing electrode. However, the structure and the control process of the electrodes are complex, and the electrodes wear easily while drilling. Besides, this method is still in the stage of simulation research. Thus, more research work on resistivity measurement in OBM should be undertaken.

Capacitively coupled contactless conductivity detection (C^4^D) technique is proposed as a contactless method for conductivity detection [20,21,22], which provides an effective approach for solving the blocking problem of non-conductive mediums. It has the advantages of low cost, simple construction, and good real-time performance [23,24,25,26,27,28]. Currently, C^4^D technique is mainly applied in the research field of analytical chemistry [20,21,22]. The inductive coupling principle is an indirect excitation method, which is generating an induced voltage in the secondary coil by applying an AC excitation signal to the primary coil [29,30,31].

By referring to the idea of the C^4^D technique and the inductive coupling principle, a new oil-based LWD method is proposed in this work [32]. On the one hand, the C^4^D technique is introduced to the well logging field to solve the electrical insulation problem of the OBM. In detail, the C^4^D technique is used to construct an effective alternating current (AC) measurement path for the measurement signal to go through the non-conductive OBM, so the influence of the electrical insulation problem of the OBM on formation resistivity measurement is overcome. On the other hand, the inductive coupling principle is used to form an induced voltage signal and implement indirect excitation of the AC measurement path. Based on the proposed method, a corresponding logging instrument will be developed, and numerical simulation and practical experiments will be carried out to verify the feasibility and effectiveness of the proposed method.

## 2. Methods

### 2.1. Application of C^4^D Technique in LWD

**Figure 1:**
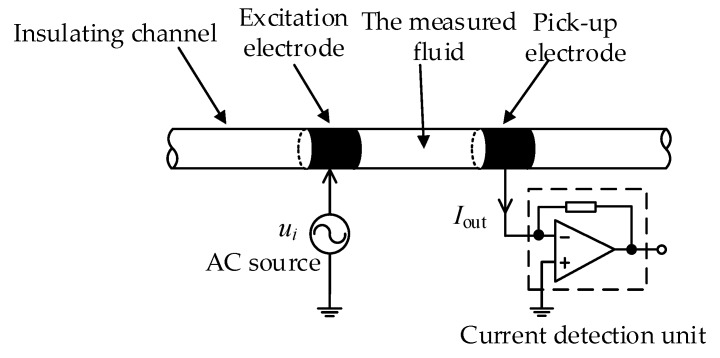
Basic structure of the C^4^D sensor.

The basic structure of the C^4^D sensor [33] is shown in Figure 1. The C^4^D sensor includes an AC source, two metal electrodes (the excitation electrode and the pick-up electrode), an insulating channel and a current detection unit.

**Figure 2:**
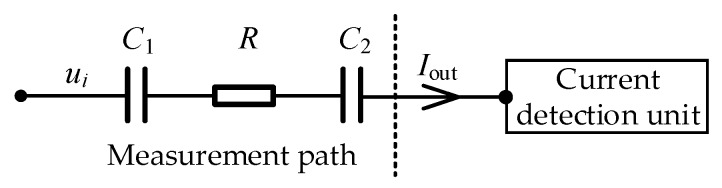
Simplified equivalent circuit of the measurement path of the C^4^D sensor.

The simplified equivalent circuit of the measurement path of the C^4^D sensor is shown in Figure 2. *C*_1_ is the coupling capacitance formed by the excitation electrode, the insulating channel, and the measured conductive fluid. *C*_2_ is the coupling capacitance formed by the pick-up electrode, the insulating channel, and the measured conductive fluid. *R* is the equivalent resistance of the measured fluid between the two electrodes. When an AC voltage *u_i_* is applied to the excitation electrode, an output current *I*_out_ will be obtained on the pick-up electrode by the current detection unit. With the obtained output current, the conductivity of the measured fluid can be obtained.

The characteristic of the C^4^D technique is to measure the conductivity of the measured object when an insulating medium exists between the electrode and the object. The measurement approach of the C^4^D technique has provided an alternative solution to solve the problem of blocking the low-frequency measurement path between electrodes and the formation caused by the non-conductive OBM.

**Figure 3:**
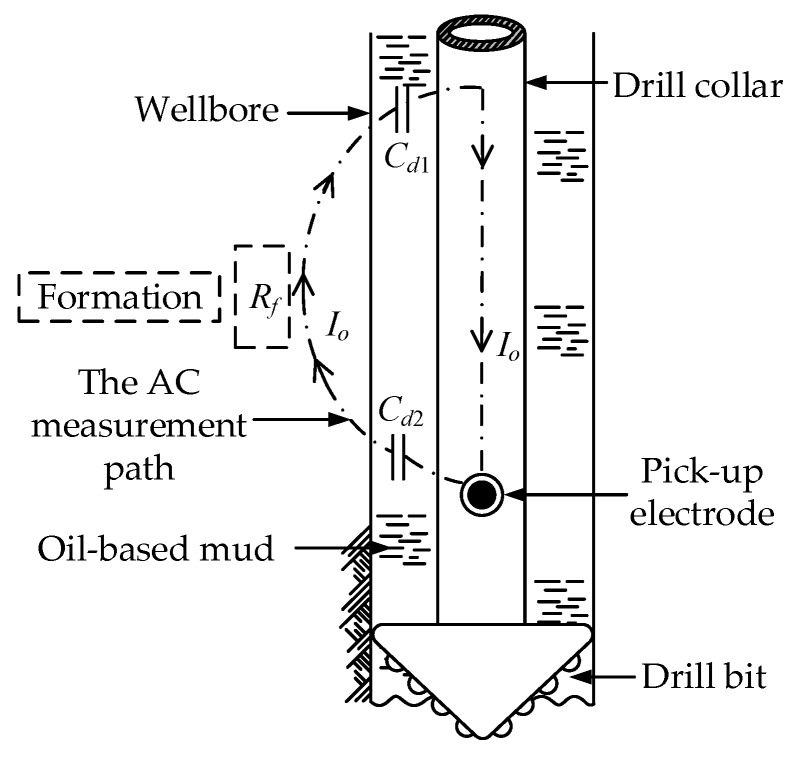
Diagram of the application of the C^4^D technique in LWD.

The diagram of the application of the C^4^D technique in LWD is shown in Figure 3. *C_d_*_1_ is the coupling capacitance formed by the drill collar, the non-conductive OBM and the formation. *C_d_*_2_ is the coupling capacitance formed by the pick-up electrode, the non-conductive OBM and the formation. *R_f_* is the equivalent resistance of the formation. The application of the C^4^D technique can overcome the electrical insulation problem of the OBM and construct an effective AC measurement path.

### 2.2. Application of Inductive Coupling Principle in LWD

**Figure 4:**
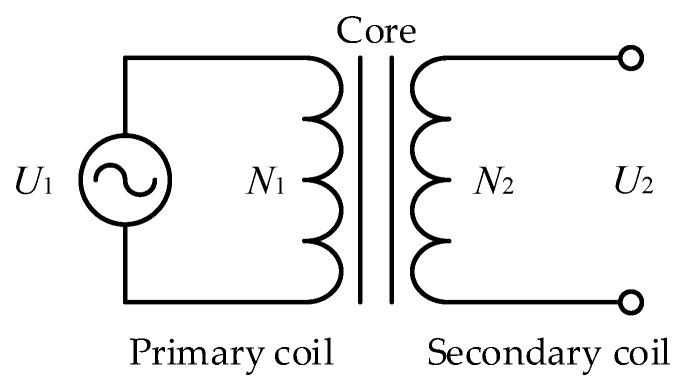
Basic principle of the inductive coupling principle.

Originally, a low-frequency excitation voltage is directly applied to the electrode system in LWD. However, the manufacturing process of electrodes is difficult and the electrodes are easy to wear while drilling, limiting the application of the direct excitation method. In 1967, researcher ARPS, J.J. applied the inductive coupling principle to LWD [29]. The basic principle of the inductive coupling principle is shown in Figure 4. Take the transformer as an example. As can be seen from Figure 4, the transformer is composed of a primary coil, a secondary coil and a core. The number of turns of the primary coil is *N*_1_ and the number of turns of the secondary coil is *N*_2_.

By applying an AC voltage *U*_1_ to the primary coil, an induced voltage *U*_2_ will be generated in the secondary coil. The induced voltage *U*_2_ is determined by the following equation:

(1) $$U_{2}=\frac{U_{1}N_{2}}{N_{1}}$$

**Figure 5:**
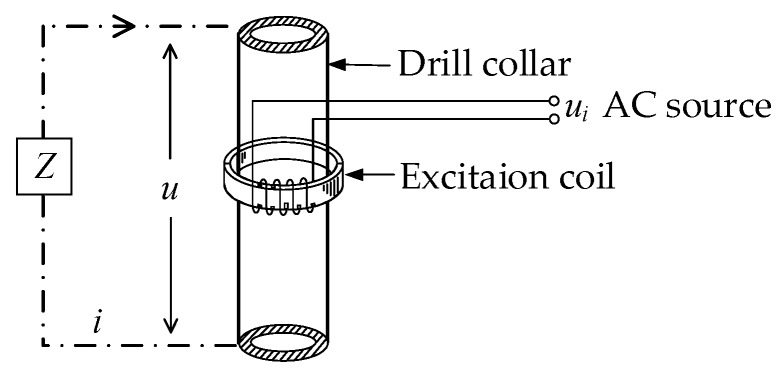
Diagram of the application of the inductive coupling principle in LWD.

Based on the inductive coupling principle, a stable induced voltage can be generated in the secondary coil of the transformer, which can be used as the excitation voltage of the logging instrument. The diagram of the application of the inductive coupling principle in LWD is shown in Figure 5. An excitation coil is introduced as the primary coil. The measurement path consisted of the drill collar, the pick-up electrodes, the OBM and the formation is regarded as the secondary coil. By applying an AC voltage to the excitation coil, an induced voltage *u* is generated at the upper and lower drill collar to provide an indirect excitation voltage for the logging instrument. *Z* is the impedance of the OBM and the formation.

The measurement path including the drill collar, the OBM and the formation can be regarded as the secondary side of a transformer with one turn. The induced voltage *u* is determined by the following equation:

(2) $$u=\frac{u_{i}}{N_{t}}$$

where *u_i_* is the voltage of the AC source, *N_t_* is the number of turns of the excitation coil.

### 2.3. The New Oil-Based LWD Method

**Figure 6:**
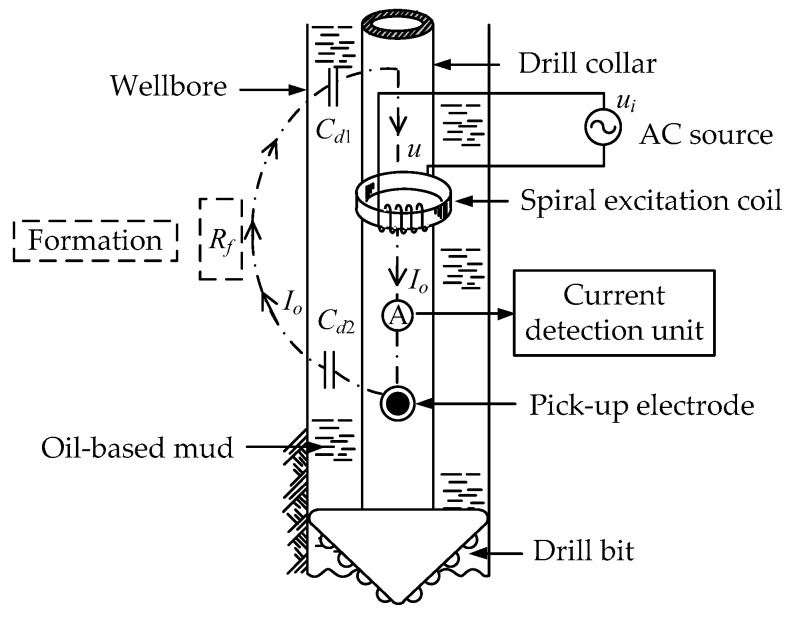
Basic structure of the logging instrument.

In order to describe the new oil-based LWD method clearly, the proposed method is applied to the development of a logging instrument. The basic structure of the logging instrument is shown in Figure 6. The logging instrument consists of an AC source, an excitation coil, a current detection unit, a pick-up electrode, a drill collar and a drill bit. The application of the C^4^D technique can overcome the electrical insulation problem of the OBM and construct an effective AC measurement path. The AC measurement path includes the drill collar, the OBM, the formation and the pick-up electrode. According to the measurement principle of the C^4^D technique, the pick-up electrode, the non-conductive OBM and the conductive formation can form a coupling capacitance, which constitutes an AC circuit path for the measurement signal to go through the OBM. In this way, the electrical insulation problem of the OBM is overcome. The inductive coupling principle is used to generate an indirect excitation source for the AC measurement path. For the whole resistivity measurement process, firstly, according to the inductive coupling principle, when an AC source is applied to the excitation coil, an induced voltage can be formed above and below the excitation coil, which is to be the excitation voltage of the AC measurement path. Secondly, the signal goes through the AC measurement path, and then a current which carries the resistivity information of the formation can be measured on the pick-up electrode by the current detection unit. Lastly, the formation resistivity can be obtained with the measured current.

**Figure 7:**
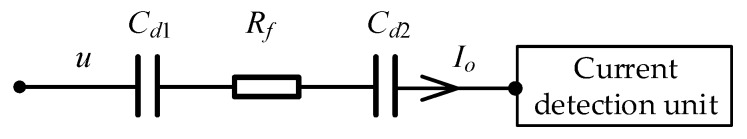
Equivalent circuit of the measurement path of the logging instrument.

**Figure 8:**
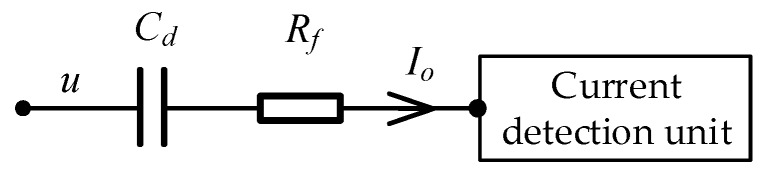
Simplified equivalent circuit of the measurement path of the logging instrument.

The equivalent circuit of the measurement path of the logging instrument is shown in Figure 7. *C_d_*_1_ is the coupling capacitance formed by the drill collar, the OBM and the formation. *C_d_*_2_ is the coupling capacitance formed by the pick-up electrode, the OBM and the formation. *R_f_* is the equivalent resistance of the formation, which is the measurement target. The simplified equivalent circuit of the measurement path of the logging instrument is shown in Figure 8. *C_d_* is the combined capacitance of *C_d_*_1_ and *C_d_*_2_.

The equivalent resistance of the formation *R_f_*, also termed the apparent resistance of the formation, can be determined by the following equation:

(3) $$R_{f}=\frac{u}{I_{o}}\cos\left(\psi \right)$$

where *u* is the induced voltage, *I_o_* is the measured current, *ψ* is the impedance angle of the impedance of the formation and the OBM.

The apparent resistivity of the formation *R_a_* is the measured resistivity of the logging instrument, which can be determined by the following equation:

(4) $$R_{a}=k\frac{u}{I_{o}}\cos\left(\psi \right)=kR_{f}$$

where *k* is the instrument constant. It is determined by the following equation:

(5) $$k=\frac{R_{a}I_{o}}{u\cos(\psi )}=\frac{R_{t}I_{o}}{u\cos(\psi )}$$

where *R_t_* is the real resistivity of the formation. The instrument constant *k* is a key parameter of the logging instrument and is affected by the size of the pick-up electrode. Generally, *k* is used to convert the measured apparent resistance of the formation to the apparent resistivity of the formation. It can be obtained by assuming that the apparent resistivity *R_a_* is equal to the real resistivity *R_t_* when the parameters such as the wellbore diameter, the OBM resistivity and the formation resistivity are known.

## 3. Results

### 3.1. Numerical Simulation

In order to verify the feasibility of the proposed method, numerical simulation of the developed logging instrument was carried out. In this part, various instrument parameters such as the measurement accuracy of formation resistivity, the detection depth and invasion radius, the influence of surrounding rock and the vertical resolution were examined to test the performance of the logging instrument.

#### 3.1.1. Numerical Simulation Setup

**Figure 9:**
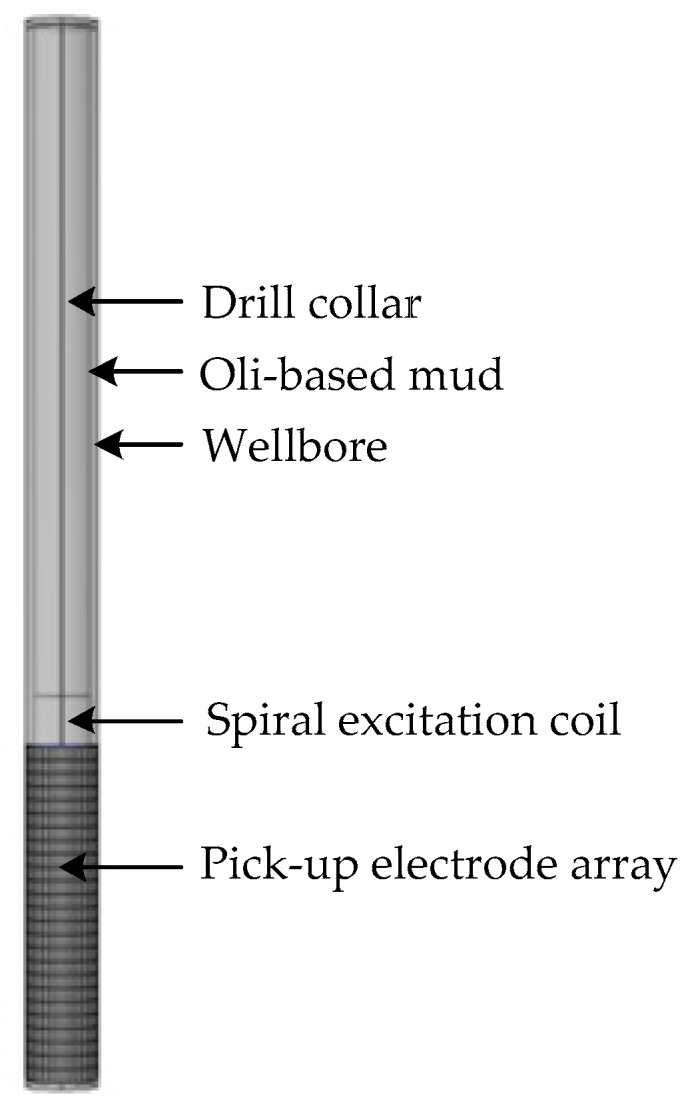
Diagram of the logging instrument.

In the well logging field, logging response is generally analyzed by numerical methods rather than analytical methods, because the logging environment is extremely complex, and all environmental factors such as wellbore, invasion zone, surrounding rock, mud cake, temperature and pressure will all affect the logging results [34,35]. In this work, the software COMSOL Multiphysics (COMSOL, Inc., Stockholm, Sweden) is used to analyze the logging response of the instrument, which is a commercial finite element modeling software in the well logging field. The logging instrument is developed in COMSOL software and the AC/DC module of the COMSOL software is used to carry out the numerical simulation. The diagram of the logging instrument is shown in Figure 9. As is shown in Figure 9, the logging instrument consists of the drill collar, the excitation coil and the detection electrode array.

**Figure 10:**
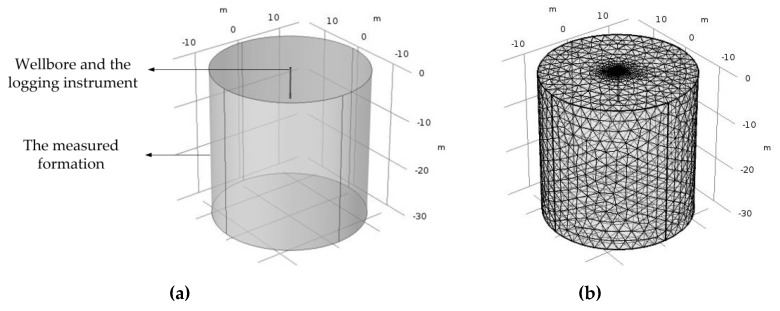
Simulation setup of the logging instrument: (**a**) setup; (**b**) meshing diagram.

The simulation setup is shown in Figure 10. There are mainly two parts, one is the wellbore and the logging instrument, and the other is the measured formation. The logging instrument is situated inside the wellbore and there is oil-based mud between the logging instrument and the wellbore. Figure 10b shows the meshing diagram of the whole area.

In COMSOL software, the finite element method (FEM) is used to analyze the logging response. The logging model needs to satisfy quasi-static electromagnetic field conditions, i.e.

(6) $$\{\begin{array}{ll} \nabla \cdot ((\sigma (x,y,z)+j\omega \varepsilon (x,y,z))\nabla \nu (x,y,z))=0 & (x,y,z)\subseteq \Pi \\ \nu_{1}(x,y,z)=U & (x,y,z)\subseteq \Gamma_{1}\\ \nu_{2}(x,y,z)=0 & (x,y,z)\subseteq \Gamma_{2}\\ \frac{\partial \nu (x,y,z)}{\partial \vec{n}}=0 & (x,y,z)\subseteq \Gamma_{0} \end{array}$$

where *σ*(*x*, *y*, *z*) is the space conductivity, *ε*(*x*, *y*, *z*)is the dielectric constant, *v*(*x*, *y*, *z*) is the potential distribution, *ω* is the angle frequency of the AC voltage, Π is the sensing field, Γ_1_ is the space location of upper drill collar, Γ_2_ is the space location of lower drill collar, Γ_0_ is the surrounding formation boundary and $\vec{n}$ is the unit normal vector of the surrounding formation boundary.

#### 3.1.2. Numerical Simulation Results

**Figure 11:**
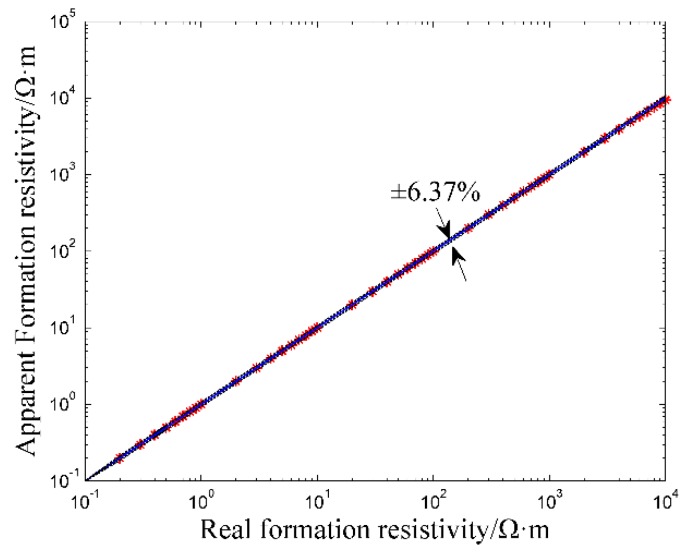
Resistivity measurement result of the logging instrument.

**Figure 12:**
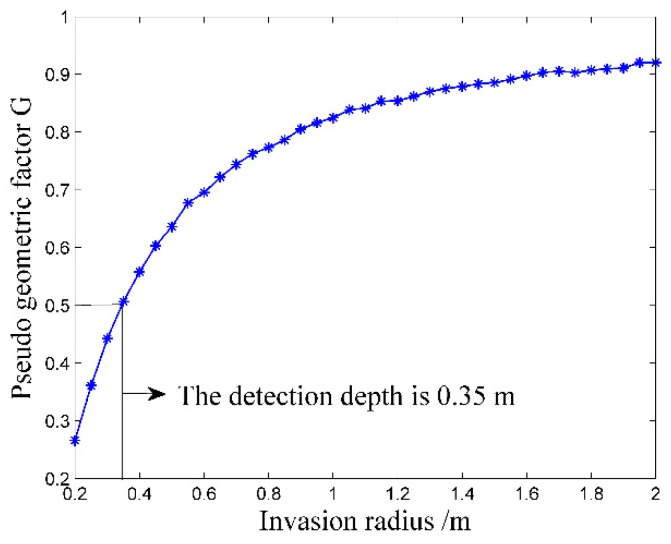
Detection depth and invasion radius.

**Figure 13:**
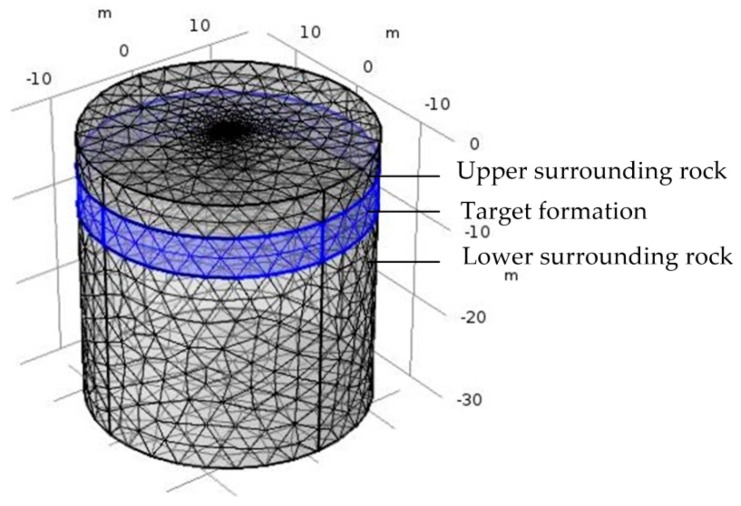
Three-layer formation model.

**Figure 14:**
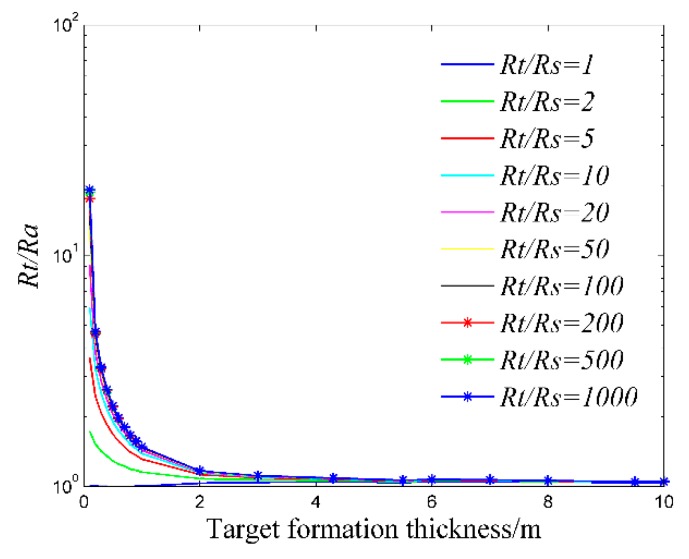
Influence of surrounding rock.

**Figure 15:**
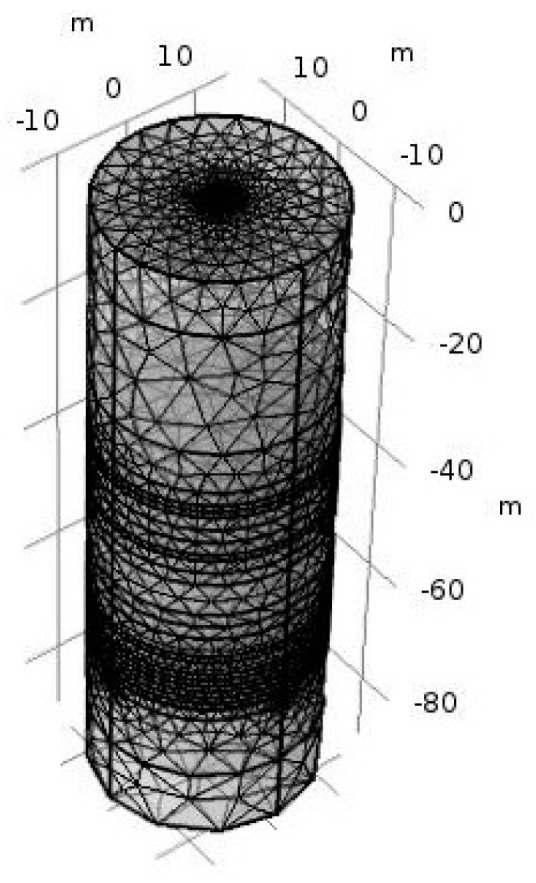
Oklahoma formation model.

**Figure 16:**
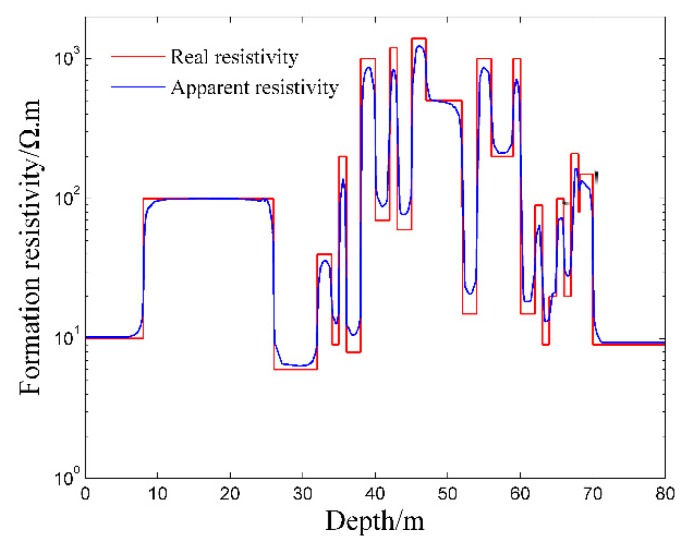
Logging response of the logging instrument to the Oklahoma formation.

1. Measurement Accuracy of Formation Resistivity
  According to the relevant published literatures, the range of oil-based logging instrument is generally between 0.2 Ω·m and 10,000 Ω·m [6,17,18]. In the numerical simulation, the formation resistivity is set between 0.2 Ω·m and 10,000 Ω·m. The relative dielectric constant of OBM is set to 3 and the resistivity of OBM is set to 1e11 Ω·m.
  In this work, the relative error is introduced to evaluate the measurement accuracy of the logging instrument. The relative error *σ_R_* can be determined by the following equation:
  (7) $$\sigma_{R}=\frac{R_{a}-R_{t}}{R_{t}}\;\times \;100\%$$
  where *R_a_* is the measured apparent resistivity of the logging instrument, *R_t_* is the real resistivity of the measured formation.
  The resistivity measurement result of the logging instrument is shown in Figure 11.
  As is shown in Figure 11, the measured apparent formation resistivity is very close to the real formation resistivity. For the range of 0.2 Ω·m to 2000 Ω·m, the maximum relative error *σ_R_* is ± 0.56% and for the range of 2000 Ω·m to 10,000 Ω·m, the maximum relative error *σ_R_* is ± 6.37%. The simulation result shows that the logging instrument has good measurement accuracy in the common range of 0.2 Ω·m to 10,000 Ω·m.
2. Detection Depth and Invasion Radius
  In the well logging field, detection depth refers to the horizontal range of the detectable formation that affects the measurement results, while invasion radius means the invasion degree of the OBM into the formation while drilling [36,37]. The detection depth of the logging instrument is generally determined by the pseudo geometric factor, i.e. the detection depth equals to the invasion radius where the pseudo geometric factor is 0.5 [36,37]. The pseudo geometric factor *G* is determined by the following equation:
  (8) $$G=\frac{R_{a}-R_{t}}{R_{x}-R_{t}}$$
  where *R_a_* is the apparent resistivity of the formation, *R_t_* is the real resistivity of the formation, *R_x_* is the apparent resistivity of the formation when the invasion radius is infinite.
  As is shown in Figure 12, the pseudo geometric factor *G* increases with the increase of the invasion radius. When the invasion radius is large enough, the pseudo geometric factor *G* is almost unchanged. At *G* = 0.5, the invasion radius is 0.35 m, so the detection depth of the logging instrument is 0.35 m.
3. Influence of Surrounding Rock and Vertical Resolution
  Influence of surrounding rock refers to the influence of upper and lower surrounding rock resistivity (*R_s_*) on the target formation resistivity (*R_t_*). The three-layer formation model is shown in Figure 13.
  Correction coefficient *R_t_*/*R_a_* is generally used to describe the influence of surrounding rock [36,37]. The closer *R_t_*/*R_a_* is to 1, the smaller the influence of surrounding rock is. As is shown in Figure 14, correction coefficient *R_t_*/*R_a_* is negatively correlated with the thickness of the target formation and positively correlated with *R_t_*/*R_s_*, which is consistent with the theory of well logging [36,37].
  Oklahoma formation model is a classic isotropic model used to test electric logging methods [36,38,39]. It has a large change in resistivity and thickness, and the thinnest formation thickness is only 0.2 m, which can fully reflect the complex situation of the real formation and evaluate the performance of the logging instrument. As is shown in Figure 15, the vertical depth of the Oklahoma formation is 80 m and the diameter of the Oklahoma formation is 30 m. In the Oklahoma formation model, the grid density reflects the thickness of the formation. The thickness of formation changes greatly, which can test the vertical resolution of the logging instrument.
  Figure 16 shows the logging response of the logging instrument to the Oklahoma formation. As is shown in Figure 16, the measured apparent resistivity of the logging instrument is close to the real formation resistivity. Further, the variation trend of the apparent resistivity is basically consistent with that of the real formation resistivity, indicating that the logging instrument can accurately reflect the resistivity change of the Oklahoma formation. Besides, the logging instrument can reflect the thickness change of the Oklahoma formation and even detect the formation with the thickness of 0.2 m at the vertical depth range of 68 m to 68.2 m, showing that the logging instrument has a high vertical resolution of thin formation layers.

### 3.2. Practical Experiments

In order to further test the performance of the logging instrument, resistance box and well logging experiments were carried out. The resistance box experiment is a principle verification experiment. The well logging experiment is carried out to evaluate the performance of the developed logging instrument.

#### 3.2.1. Resistance Box Experiment

**Figure 17:**
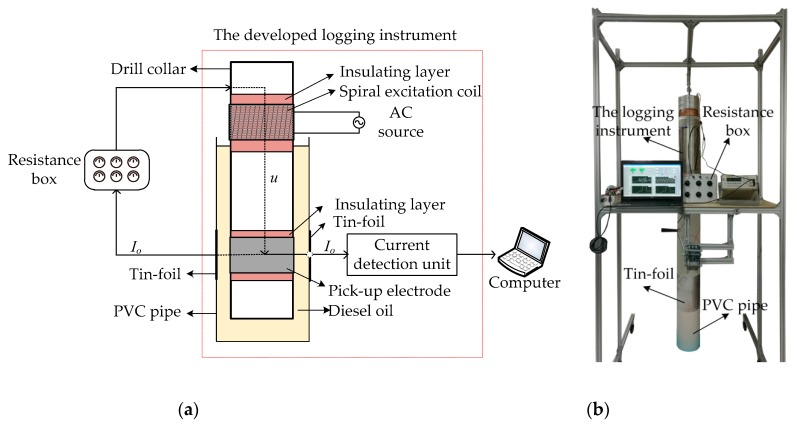
Experimental setup of resistance box experiment: (**a**) schematic; (**b**) photo.

As the resistance box experiment is a principle verification experiment, the parameters of the logging instrument such as the excitation frequency, electrode shape and size are not optimized. In this part, a resistance box is chosen to simulate the formation and diesel oil is used to simulate the OBM. In order to obtain a larger current, a circular pick-up electrode is used without considering the azimuth recognition ability. The experimental setup of resistance box experiment is shown in Figure 17. The experimental setup includes the developed logging instrument, a resistance box and a computer. The equivalent circuit of the measurement path of the logging instrument is the same as that of Figure 7.

The excitation frequency is set to 20 kHz. The resistance of the resistance box ranges from 2 kΩ to 30 kΩ. Again, the relative error of resistance measurement is used to present the results. The relative error *σ_r_* can be determined by the following equation:

(9) $$\sigma_{r}=\frac{r_{a}-r_{t}}{r_{t}}\;\times \;100\%$$

where *r_a_* is the measured apparent resistance by the logging instrument, *r_t_* is the value of the resistance box.

**Figure 18:**
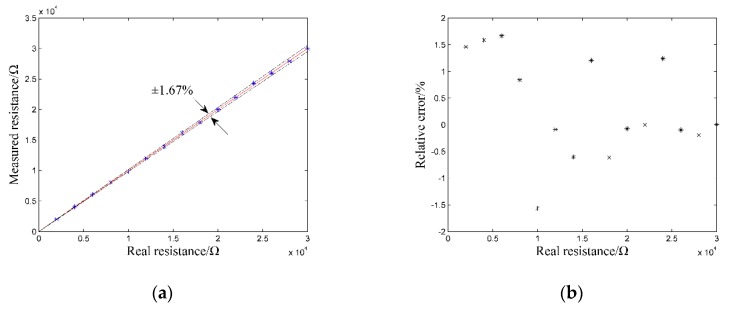
Results of resistance box experiment: (**a**) measured resistance; (**b**) relative errors.

Figure 18 shows the results of the resistance box experiment. As is shown in Figure 18, the relative errors σ*_r_* of the resistance measurement by the developed instrument are less than ± 1.67%. This result indicates that the logging instrument has good resistance measurement accuracy.

#### 3.2.2. Well Logging Experiment

**Figure 19:**
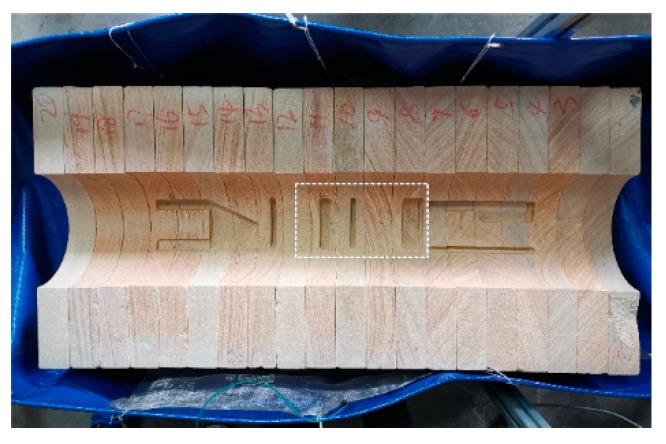
Experimental well.

**Figure 20:**
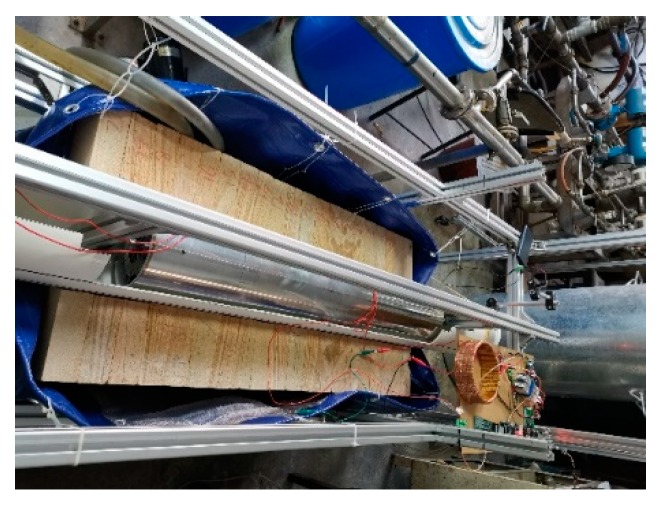
Experimental setup of well logging experiment.

Considering the real logging conditions, an experimental well is built to simulate the real formation. The experimental well is composed of sandstone, which is one of the common rocks in the real formation. The sandstones are provided by the Huiyu stone company, Hangzhou, China and the origin of the sandstones is Chengdu, China. The resistivity of the sandstones is about 4000 Ω·m. There are several artificial patterns on the wellbore of the experimental well. In addition, silicone oil is used to simulate the OBM instead of diesel oil, because it is more environmentally friendly than diesel oil. Though the excitation frequency should be as high as possible in order to reduce the capacitive reactance of the OBM, the excitation frequency is set to 50 kHz due to the limitation of the excitation coil. In order to test the azimuth recognition ability of the logging instrument, a button pick-up electrode is used in the experiment instead of the circular electrode. The developed experimental well is shown in Figure 19 and the experimental setup is shown in Figure 20.

**Figure 21:**
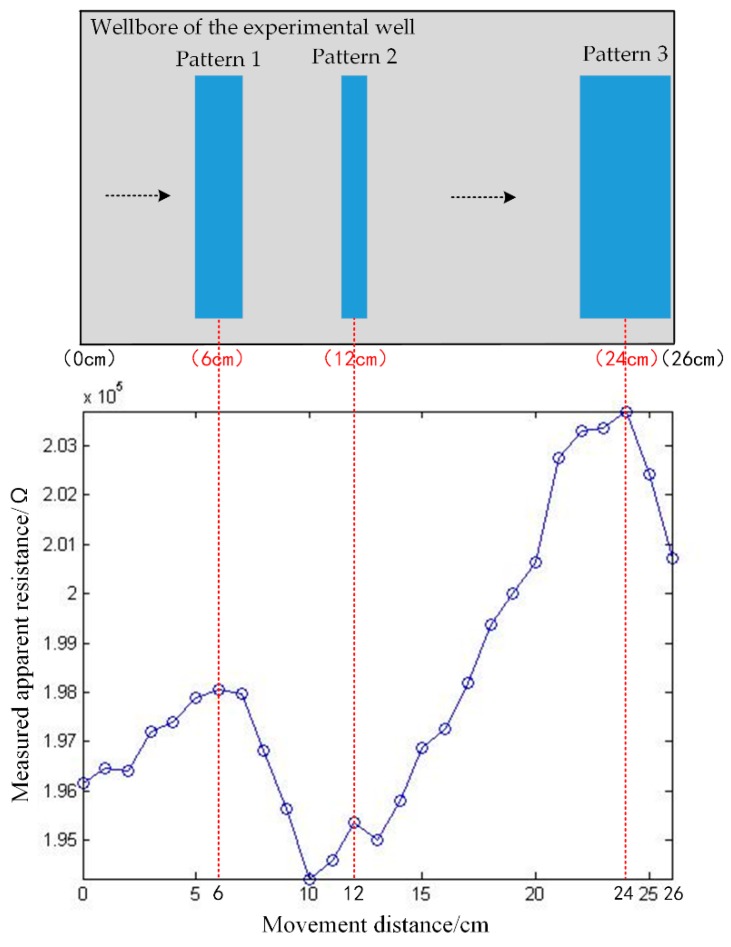
Logging response of the instrument in well logging experiment.

In this work, part of the experimental well with the length of 26 cm is tested and three patterns on the wellbore are to be measured within this range. This experiment is to investigate the logging response of the instrument concerning the measured patterns, which is shown in the dotted box in Figure 19. The three patterns are rectangular with the same length and different widths, and are placed at different positions along the axial direction of the drill collar. The diagram of the measured patterns is shown in Figure 21. Different patterns introduce air gaps of different sizes at different positions. Due to the air gap, the resistivity of the pattern areas is higher than other areas in the experimental well. During experiment, the pick-up electrode is moved by moving the drill collar. The experimental result is shown in Figure 21.

As is shown in Figure 21, the measured resistance increases when the pick-up electrode moves toward the patterns, and decreases when the electrode moves away from the patterns. It is found that there are three peaks at the movement distances of 6 cm, 12 cm and 24 cm, which are consistent with the center positions of the three patterns. Besides, as the three patterns have different widths, the widths of the three peaks are also different. The experimental result shows that the logging instrument can accurately reflect the positions of the three patterns, further indicating the feasibility of the proposed logging method.

## 4. Conclusions

Our study focuses on the measurement of the formation resistivity in the oil-based mud environment. A new oil-based LWD method is proposed by referring to the idea of the C^4^D technique and combining with the inductive coupling principle, which can solve the electrical insulation problem of the OBM and make the measurement of the formation resistivity feasible. Based on the proposed method, a corresponding logging instrument is developed and its performance is investigated by both numerical simulation and practical experiments. Numerical simulation results show that the logging instrument has good measurement accuracy in a wide measurement range of the formation resistivity. The maximum relative error of resistivity measurement in the common range of 0.2 Ω·m to 2000 Ω·m is ± 0.56%. Moreover, the logging instrument has deep detection depth and high vertical resolution, and can accurately reflect the resistivity change of the Oklahoma formation. The results of resistance box experiment show that the logging instrument has good resistance measurement accuracy and the maximum relative error is ± 1.67%. The results of well logging experiment show that the logging instrument can accurately reflect the positions of the patterns on the wellbore of the experimental well. Both numerical simulation and practical experiments verify the feasibility and effectiveness of the new method.

Compared with the conventional methods, such as making OBM conductive, using a knife-like electrode to cut through the mud cake and four-terminal measurement, the proposed method has the advantages of low cost, simple construction, and good real-time performance.

Useful knowledge and experience concerning the oil-based LWD have been obtained in this work. The research results can provide useful reference and guidance for further lateral resistivity measurement of oil-based LWD and design of logging instruments. However, the proposed method also has some limitations at the current stage. According to the measurement principle of C^4^D, the existence of the two coupling capacitances makes the measurement possible, but only the resistance of the formation is the useful information and the coupling capacitances are the background signal. In further research, the influence of the coupling capacitances needs to be either reduced by increasing the excitation frequency or canceled by introducing effective methods like the impedance elimination method and the series resonance method. In addition, the resistivity measurement is easily affected by the experimental temperature. Thus, temperature compensation should be considered in the future to overcome the influence of environmental temperature on the resistivity measurement.
